# Evolving the aims and scope of Disease Models & Mechanisms

**DOI:** 10.1242/dmm.053267

**Published:** 2026-10-30

**Authors:** Kirsty Hooper, E. Elizabeth Patton

**Affiliations:** ^1^ The Company of Biologists, Bidder Building, Station Road, Histon, Cambridge CB24 9LF, UK; ^2^ MRC Human Genetics Unit, and CRUK Scotland Centre and Edinburgh Cancer Research, Institute of Genetics and Cancer, Western General Campus, The University of Edinburgh, Edinburgh EH4 2XU, UK

## Abstract

**Summary:** DMM redefines its aims and scope to welcome disease research in all model systems, with a renewed focus on human disease models.

Disease Models & Mechanisms (DMM) was established in 2008 in response to the growing community of researchers using experimentally tractable model organisms to investigate the mechanisms underlying human disease. An increasing number of laboratories were developing disease models across diverse species; these efforts were generating significant biological and translational insights. In response, The Company of Biologists proposed the creation of a journal specifically focused on the application of model systems, particularly model organisms, to the study of human disease. From the outset, the intention was not to establish another broad molecular medicine journal in an already crowded publishing landscape. Instead, DMM was conceived as a platform to support and help define an emerging interdisciplinary field linking basic scientists and clinicians. The Company of Biologists, a not-for-profit publisher run by biomedical scientists and committed to reinvesting revenues into the scientific community, launched DMM primarily to address this clear scientific need rather than to pursue commercial objectives – its creation was fundamentally motivated by its potential value to the research community.

More recently, we have seen research and funding increasingly directed towards new-approach methodologies and non-animal technologies to model and investigate human disease. These innovative systems are high-throughput, highly translational and align with guiding principles, such as the 3Rs (‘Replacement, Reduction and Refinement’). In line with the development of these advanced systems, the scope of the journal has naturally evolved too, expanding to encompass next-generation model systems such as induced pluripotent stem cells and other stem-cell-based models, organoids, assembloids, tumouroids, organ-on-a-chip systems, primary and patient-derived tissue and cells, and computational and *in silico* approaches. Our recent Special Issue, entitled ‘*In Vitro* Models of Human Disease to Inform Mechanism and Drug Discovery’ (Hooper et al., 2026), showcased this commitment; we continue to publish in this area in our growing subject collection ‘Advanced*In Vitro* Models’.

This shift in focus from model organisms to model systems, however, does not diminish our support for the classical model organisms (vertebrate and invertebrate) that remain essential for biomedical research (Bogani et al., 2026), as well as for non-conventional large-animal and natural animal models. Moving forward, this diverse spectrum of model systems can be used as a complementary toolkit to interrogate the mechanisms of human disease and inform its diagnosis and treatment. Overall, we encourage researchers to tailor their choice of model system(s) to the biological questions they aim to address.

In the past 5 years, our Editor team and Editorial Advisory Board have expanded to include researchers working on emerging areas of research, including the use and development of non-animal models. We have also expanded the scope of our review-type articles into these emerging areas, exploring innovative model systems (Eskow and Hernando, 2025; Guallar-Garrido and Soldati, 2024; Tian et al., 2025; Xu and Prigione, 2025), techniques (Hernández-Morán et al., 2024; Ma et al., 2024) and therapeutic approaches (Adams et al., 2024; Fox and Booth, 2024; Kucinski et al., 2024; Pfeil et al., 2024; Traore et al., 2025). DMM continues to cover the full breadth of human disease, and we often signpost areas of disease biology research that we are increasingly interested in, such as how environmental exposures drive disease in the mini-focus ‘A Focus on Forever Chemicals’ (DeWitt et al., 2025; Jass et al., 2025; Yoder, 2025).

With the rapidly progressing repertoire of cutting-edge disease models and techniques being employed to study human disease, we have been receiving more high-quality papers describing the development of tools, resources, methods and protocols. To reflect this influx of papers, our Editor team relaunched our Resources & Methods article type in 2022 to encompass new disease models (that are validated to be reproducible and disease relevant), novel methods, substantial advances of an existing method, protocols and new resources of significant value to disease-biology research (Box 1). These articles are published alongside our Research Articles, which investigate the mechanisms of disease, with the aim of informing new diagnostic or therapeutic approaches in clinical research (Box 1). Both article types are celebrated in our annual Outstanding Paper Prize, awarded to the first author(s) of the winning articles. Furthermore, across all our article types, DMM is especially interested in promoting Rigour and Reproducibility in research. For instance, in an Editorial article, our Editorial Board Member Joseph Wu analysed and discussed the International Society for Stem Cell Research (ISSCR)’s guidelines to improve rigour and reproducibility in stem-cell-derived disease modelling (Juguilon and Wu, 2024). In another Editorial, our former Editor-in-Chief Monica Justice stressed the importance of including, analysing and reporting sex as a biological variable in preclinical research (Justice, 2024).

Box 1. Research and Resources & Methods articles **Research Articles**

Research Articles report new mechanistic insight into human disease using any disease model system. DMM also publishes studies that identify or evaluate candidate interventions, including drug and target screens, and gene/cell therapies.

**Resources & Methods**

Resources & Methods articles describe new disease models, novel methods, substantial advances of an existing method, protocols or new resources of significant value to disease biology research. We are especially interested in methods, tools and standards that improve rigour and reproducibility. Resources & Methods articles should clearly provide new insight into disease.

- New model systems should demonstrate their usefulness to improve our understanding of disease biology and/or to evaluate treatment approaches.
- Papers describing a new dataset or resource should explain how it will be of value to disease biology research; deposition of the resource described in a suitable repository is mandatory.
- Genome editing, lineage tracing, drug screening, chemical biology, imaging, microscopy, bioinformatics and software tools, databases, and reproducibility and validation frameworks for disease models are within the scope of Resources & Methods articles.
- Methods and protocols should be described in sufficient detail to be readily replicated in other laboratories and validated to demonstrate relevance.

Both Research and Resources & Methods articles use any disease model system and can cover any area of human disease research, including, but not limited to, the following:

- Cancer and tumour biology (Alissa et al., 2024; Diaz et al., 2024; Ginzel et al., 2025; Maggiore et al., 2025; May et al., 2025).
- Neurodegenerative (Shukla et al., 2024), neurological and psychiatric disorders.
- Metabolic disorders (Gonzalez et al., 2025; Zang et al., 2024).
- Cardiovascular disease and stroke (Eley et al., 2024; Fullenkamp et al., 2024).
- Developmental and rare genetic disorders (Desingu Rajan et al., 2024; Griffin et al., 2025; Medeiros et al., 2024; Reyes-Nava et al., 2025).
- Infectious disease and host–pathogen interaction (Ektnitphong et al., 2025; Link et al., 2024; Tapia et al., 2025; Thornton et al., 2025; Yamamoto et al., 2025).
- Musculoskeletal (Karimjee et al., 2025), renal (Jafree et al., 2025), liver (Khan et al., 2024; Wu et al., 2025), lung (Pinte et al., 2025), gastrointestinal (Hurcomb et al., 2025) and eye (Ashimori et al., 2024; Katschke et al., 2025) disease.
- Blood disorders, autoimmunity and inflammation (Scheurer et al., 2024).
- Environmental exposure (toxicants, diet, lifestyle, climate) and how it drives disease (Cabrera et al., 2026; Wallén et al., 2025).
- Regenerative medicine (Dinwoodie et al., 2024; Kaliya-Perumal et al., 2024; Kramer et al., 2024).

With our founding principles in mind, DMM continues to serve our research community as it adapts to changes in the scientific and regulatory landscape. We are purposefully model agnostic and support rigorous and reproducible research into all human diseases, using whichever tools and systems are the best fit for the job. As all our content is Open Access, it is freely available to our communities of basic and clinical researchers, health professionals, patients, and their advocates and families to help foster cross-disciplinary collaboration.

**Figure:**
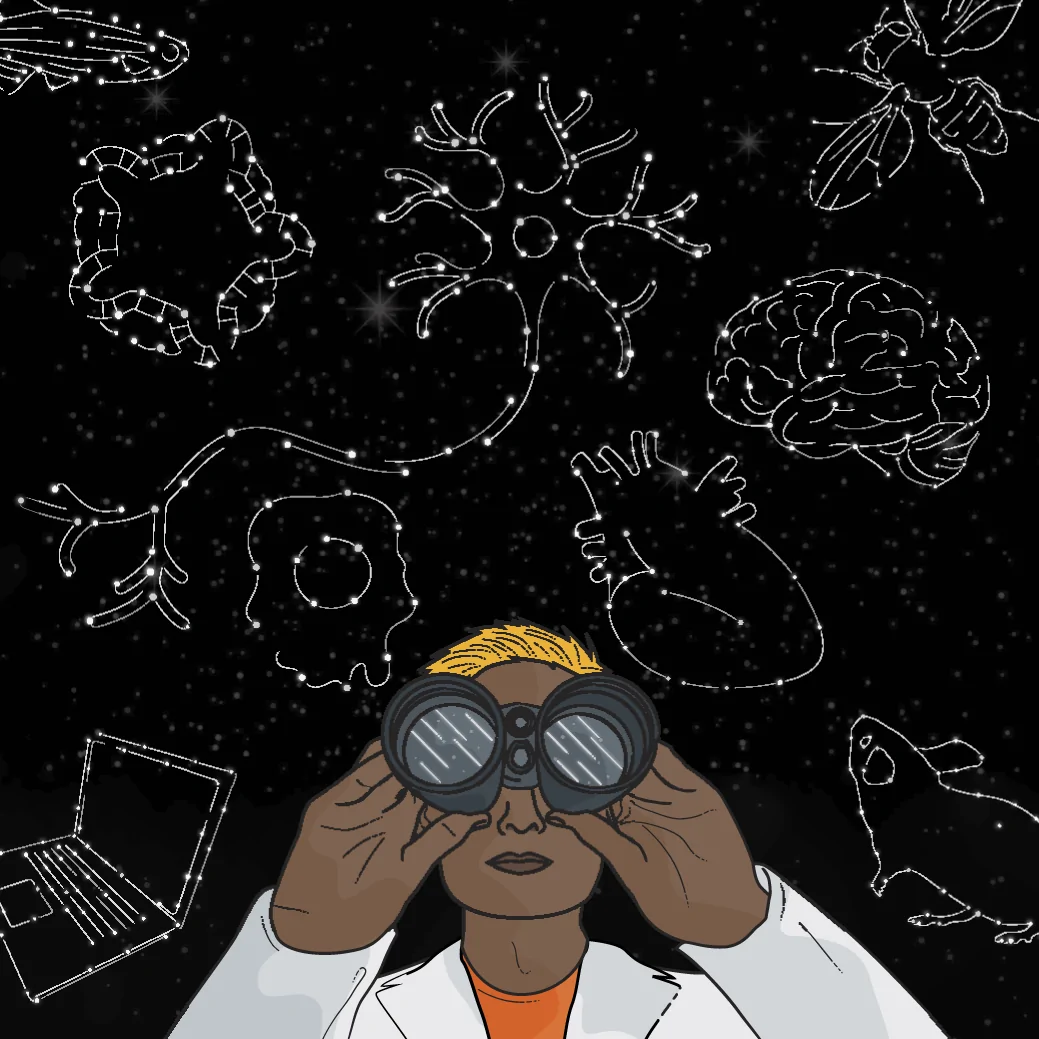
This image is by neilsmithillustration.co.uk and published under the CC-BY 4.0 license for this article.
